# Fasciculation frequency is a questionable biomarker for motor unit loss in amyotrophic lateral sclerosis

**DOI:** 10.1093/braincomms/fcaa188

**Published:** 2020-12-11

**Authors:** Josef Finsterer, Fulvio A Scorza

**Affiliations:** 1 Klinik Landstrasse, Messerli Institute, Vienna, Austria; 2 Disciplina de Neurociência, Universidade Federal de São Paulo/Escola Paulista de Medicina (UNIFESP/EPM), São Paulo, Brasil

With interest, we read the article by Bashford *et al*. (2020) about a study of 20 patients with amyotrophic lateral sclerosis (ALS) and 5 patients with benign fasciculation syndrome for the frequency of fasciculations by means of high-density surface electromyography of the brachial biceps and gastrocnemius muscles (Bashford *et al*., 2020). It was found that the frequency of fasciculations was 10 respectively 40 times higher in strong respectively weak biceps muscles of ALS patients as compared to controls (Bashford *et al*., 2020). It was concluded that fasciculation frequency as measured by high-density surface electromyography could serve as a disease biomarker of motor unit loss in ALS (Bashford *et al*., 2020). We have the following comments and concerns.

A first shortcoming of the study is that fasciculations are not unique to ALS but may occur in a number of other types of neurological conditions, particularly other types of motor neuron disease (spinal muscular atrophy, bulbospinal muscular atrophy and Allgrove syndrome), hereditary neuropathies, spinocerebellar ataxias, certain primary myopathies and other conditions (e.g. Huntington’s disease, Rett syndrome, Fabry’s disease, Gerstmann–Sträussler disease or GM-2-gangliosidosis) (Finsterer and Aliyev, 2015). Since benign fasciculation syndrome may include a number of unidentified genetic disorders, it would be more appropriate to compare ALS patients rather with patients with spinal muscular atrophy than with benign fasciculation syndrome.

A second shortcoming of the study is that surface-electromyography was applied to record fasciculations. Surface-electromyography recording may miss fasciculations which originate from deeper parts of the muscle why a quantitative assessment of the fasciculations by means of high-density surface electromyography may provide false-negative results. Thus, the provided data not necessarily represent the exact quantities of fasciculations.

Furthermore, it is unclear how many ALS patients with the bulbar onset and how many with limb onset were included in the study. This point is crucial as patients with bulbar onset may not present with extensive fasciculations in limb muscles during the initial period of the disease.

A further shortcoming of the study is that it was not reported how many of the 20 included patients were under a treatment with riluzole and how many of these did not receive this treatment. Knowing the number of patients receiving riluzole is crucial as it may strongly influence the quantity of fasciculations (Hugon, 1996).

Disease duration was highly variable among the 20 ALS patients, ranging from 11 to 60 months (Bashford *et al*., 2020). Since the frequency of fasciculations may strongly relate to disease duration (Tsugawa *et al*., 2018), it should be calculated if fasciculation frequency correlated with disease duration or not.

There is no explanation provided why fasciculation frequency in strong biceps muscles increased whereas fasciculation frequency declined in strong gastrocnemius muscles within the observational period of 14 months.

Overall, the presented study has a number of shortcomings that should be addressed before drawing conclusions as those provided. The investigated cohort should be more homogeneous with regard to ALS type, disease duration and treatment and should be compared to a homogenous disease control group.

## Data availability

Data sharing is not applicable to this article as no new data were created or analysed.

## Competing interests

The authors report no competing interests.
